# Excessive Laughter-like Vocalizations, Microcephaly, and Translational Outcomes in the *Ube3a* Deletion Rat Model of Angelman Syndrome

**DOI:** 10.1523/JNEUROSCI.0925-21.2021

**Published:** 2021-10-20

**Authors:** Elizabeth L. Berg, Shekib A. Jami, Stela P. Petkova, Annuska Berz, Timothy A. Fenton, Jason P. Lerch, David J. Segal, John A. Gray, Jacob Ellegood, Markus Wöhr, Jill L. Silverman

**Affiliations:** ^1^MIND Institute and Department of Psychiatry and Behavioral Sciences, University of California Davis School of Medicine, Sacramento, California 95817; ^2^Department of Neurology, Center for Neuroscience, University of California Davis, Davis, California 95618; ^3^Behavioral Neuroscience, Experimental and Biological Psychology, Faculty of Psychology, Philipps-University Marburg, Marburg, 35032, Germany; ^4^Center for Mind, Brain and Behavior, Philipps-University Marburg, Marburg, 35032, Germany; ^5^Mouse Imaging Centre, Hospital for Sick Children, Toronto, Ontario M5T 3H7, Canada; ^6^Department of Medical Biophysics, University of Toronto, Toronto, Ontario M5S, Canada; ^7^Wellcome Centre for Integrative Neuroimaging, University of Oxford, Oxford, OX3 9DU, United Kingdom; ^8^MIND Institute, Genome Center, and Department of Biochemistry and Molecular Medicine, University of California Davis, Davis, California 95616; ^9^KU Leuven, Faculty of Psychology and Educational Sciences, Research Unit Brain and Cognition, Laboratory of Biological Psychology, Social and Affective Neuroscience Research Group, Leuven, B-3000, Belgium; ^10^KU Leuven, Leuven Brain Institute, Leuven, B-3000, Belgium

**Keywords:** Angelman syndrome, behavior, play, rat, Ube3a, ultrasonic vocalization

## Abstract

Angelman syndrome (AS) is a rare genetic neurodevelopmental disorder characterized by intellectual disabilities, motor and balance deficits, impaired communication, and a happy, excitable demeanor with frequent laughter. We sought to elucidate a preclinical outcome measure in male and female rats that addressed communication abnormalities of AS and other neurodevelopmental disorders in which communication is atypical and/or lack of speech is a core feature. We discovered, and herein report for the first time, excessive laughter-like 50 kHz ultrasonic emissions in the *Ube3a*^mat–/pat+^ rat model of AS, which suggests an excitable, playful demeanor and elevated positive affect, similar to the demeanor of individuals with AS. Also in line with the AS phenotype, *Ube3a*^mat–/pat+^ rats demonstrated aberrant social interactions with a novel partner, distinctive gait abnormalities, impaired cognition, an underlying LTP deficit, and profound reductions in brain volume. These unique, robust phenotypes provide advantages compared with currently available mouse models and will be highly valuable as outcome measures in the evaluation of therapies for AS.

**SIGNIFICANCE STATEMENT** Angelman syndrome (AS) is a severe neurogenetic disorder for which there is no cure, despite decades of research using mouse models. This study used a recently developed rat model of AS to delineate disease-relevant outcome measures to facilitate therapeutic development. We found the rat to be a strong model of AS, offering several advantages over mouse models by exhibiting numerous AS-relevant phenotypes, including overabundant laughter-like vocalizations, reduced hippocampal LTP, and volumetric anomalies across the brain. These findings are unconfounded by detrimental motor abilities and background strain, issues plaguing mouse models. This rat model represents an important advancement in the field of AS, and the outcome metrics reported herein will be central to the therapeutic pipeline.

## Introduction

Angelman syndrome (AS) is a rare neurodevelopmental disorder characterized by intellectual disability, impaired communication, ataxia, seizures, as well as a happy disposition with a high degree of excitability, smiling, and easily provoked laughter (Williams and Franco, 2010; Bird, 2014). AS is caused by dysfunction of maternal ubiquitin protein ligase E3A (UBE3A), typically from a *de novo* deletion in the 15q11-q13 region (Albrecht et al., 1997). Restoring functional UBE3A is seemingly possible by innovative gene therapy approaches, including antisense oligonucleotides (Meng et al., 2015), viral vector delivery (Daily et al., 2011), artificial transcription factors (Bailus et al., 2016), stem cell-mediated therapies (Adhikari et al., 2021), and the cutting edge Cas9 (Wolter et al., 2020). Gene replacement therapy is therefore on the horizon for AS; and indeed, two clinical trials using “gene therapy-like” antisense oligonucleotide interventions began recruitment in 2020 (GeneTx NCT04259281; Roche NCT04428281).

Indispensable to such a strategy of therapeutic development are *in vivo* studies using preclinical model systems with rigorous translational outcomes. One domain that is critically impaired in AS and other neurodevelopmental disorders but difficult to study in preclinical models because of their lack of human-interpretable language is communication. The increasing availability of rat models of neurodevelopmental disorders opens up new opportunities to develop preclinical outcome measures of social communication. While the mouse has been the preferred model species in recent decades because of the genetic technologies available, there are complex behaviors and physiological processes difficult or impossible to investigate in mice that are easily observable in rats (Hofer et al., 2002; Portfors, 2007; Hammerschmidt et al., 2012; Wöhr and Schwarting, 2013; Portfors and Perkel, 2014; Ellenbroek and Youn, 2016).

One prominent example is the greater sophistication and complexity in the rat acoustic communication system. While both mice and rats emit ultrasonic vocalizations (USVs), rats emit USVs that serve as situation-dependent, evolved signals which accomplish important communicative functions that are not observed as functions of mouse USV, such as low-frequency 22 kHz “alarm calls,” which rats use to warn of potential threats (Blanchard et al., 1991; Wöhr and Schwarting, 2007, 2013; Sadananda et al., 2008; Brudzynski, 2013; Kisko et al., 2017; Fendt et al., 2018). The recent generation of the first rat model of AS therefore provides the unique opportunity to study a greater diversity of social and communication behaviors compared with those previously available in mouse models (Jiang et al., 2010; Huang et al., 2013; Parker et al., 2014; Ellenbroek and Youn, 2016; Harony-Nicolas, 2017; Homberg et al., 2017; Kisko et al., 2018; Kondrakiewicz et al., 2019; Dutta and Crawley, 2020; Netser et al., 2020; Reppucci et al., 2020).

To build on the initial reports describing the *Ube3a* deletion rat model of AS, which revealed deficits in motor, cognition, social approach, and pup vocalizations (Berg et al., 2020c; Dodge et al., 2020), we sought to investigate nuanced social behaviors and further characterize vocalization patterns. With numerous novel therapies being assessed in clinical trials and at the investigational drug discovery level, AS-relevant outcome measures are vital for demonstrating functional efficacy of the varied intervention approaches. Leveraging the rat's social communication system, we discovered that the *Ube3a* maternal deletion rat (*Ube3a*^mat–/pat+^) produced excessive signals of positive affect characteristic of AS. Several other AS-relevant phenotypes were evident, including atypical social interactions and maladaptive impairments in gait and cognition. We also identified reduced hippocampal long-term potentiation (LTP), observed in mouse models of AS but not yet in rats, as a putative cellular mechanism underlying the learning and memory deficits apparent in the model. Finally, our neuroimaging analysis revealed decreased brain volume and pronounced increasing severity with age.

## Materials and Methods

### Subjects

Subjects were male and female Sprague Dawley *Ube3a*^mat–/pat+^ rats and their WT littermates (*Ube3a*^mat+/pat+^) generated from breeding pairs of paternal *Ube3a* deletion females and WT males purchased from Envigo. The initial generation of *Ube3a* deletion rats using CRISPR/Cas9 was described previously (Berg et al., 2020c). Genotyping was performed using a small sample of tail tissue collected at postnatal day (PND) 2, REDExtract-N-Amp (Sigma Aldrich), and primers Rube1123 TAGTGCTGAGGCACTGGTTCAGAGC, Rube1606r TGCAAGGGGTAGCTTACTCATAGC, Ub3aDelSpcfcF6 ACCTAGCCCAAAGCCATCTC, and Ub3aDelR2 GGGAACAGCAAAAGACATGG. All animals were socially housed in a temperature-controlled vivarium maintained on a 12:12 light-dark cycle with testing occurring during the light phase. All procedures were conducted in compliance with the National Institutes of Health's *Guide for the care and use of laboratory animals* and approved by the Institutional Animal Care and Use Committee of the University of California Davis. To minimize the carryover effects from repeated testing and handling, seven mixed-sex cohorts of rats were tested and behavioral tests were conducted in order of least to most stressful with at least 48 h break between tests. Each cohort was comprised of 4-9 litters, and subjects were sampled as follows: subjects for 50 kHz USV playback were sampled from Cohort 1; contextual and cued fear conditioning from Cohort 2; gait analysis, heterospecific play, and social play from Cohort 3; acoustic startle and LTP from Cohort 4; spontaneous exploratory USV from Cohort 5; spontaneous alternation from Cohort 6; and olfactory discrimination from Cohort 7. Following behavioral testing, rats from Cohort 3 were perfused for MRI.

### Juvenile USV in response to heterospecific play

At PND 30 to PND 34, rats were provided daily heterospecific play sessions involving manual stimulation using a slightly abbreviated procedure from those described previously (Burgdorf and Panksepp, 2001; Schwarting et al., 2007; Wöhr et al., 2009). For 5 min on 5 consecutive days, rats were individually manipulated by a familiar experimenter using a single clean hand within a clean, empty version of the home cage with fresh bedding (37.2 cm [length] × 30.8 cm [width] × 18.7 cm [height]; illuminated to ∼30 lux) while vocalizations were recorded with an overhead ultrasonic microphone (Avisoft Bioacoustics) for later scoring by a trained observer blinded to genotype. The number of calls emitted during each 30 s interval were counted and classified as either high (50 kHz) or low (short 22 kHz) frequency using a threshold of 33 kHz. Calls emitted during the minute immediately preceding the heterospecific play sessions on days 2-4 (“anticipation”) were also counted and classified.

All rats were handled by the experimenter in a standardized fashion (5 min on 3 d) before the first heterospecific play session. The physical manipulations performed during heterospecific play were tickling the subject's neck (2×), tickling the subject's belly (1×), pushing into their shoulders (“push and drill”; 1×), and flipping the subject onto their back and momentarily pinning them down (“flip over”; 3×). Each manipulation lasted 30 s with three 30 s breaks interspersed at 0, 60, and 150 s, during which the experimenter did not initiate touching the subject but moved their hand around the cage to encourage following or chasing. To provide a standardized experience, a single experimenter conducted the procedure for all subjects and the experimenter remained unaware of USV being emitted during the test, performing the manipulations in an equivalent manner for all rats. To mitigate any potential effect of order, the sequence of manipulations was reordered each day but remained consistent across all animals. The testing order of the subjects was also changed from day to day.

### Juvenile spontaneous exploratory USV

At PND 30, rats were individually placed in a clean, empty version of the home cage (illuminated to ∼30 lux) with clean bedding for 5 min similarly to methods described previously (Schwarting et al., 2007; Wöhr et al., 2008). Recording of USVs began immediately following the subject being placed into the cage, and no other animals or any experimenter were present in the room during recording. Calls were classified by a trained observer blinded to genotype as either high (50 kHz) or low (short 22 kHz) frequency using a threshold of 33 kHz.

### Juvenile USV in response to playback of 50 kHz USV

At PND 30 ± 4, subjects were individually presented with 1 min of natural prosocial 50 kHz USV while on a radial maze illuminated to ∼8 lux as described previously (Berg et al., 2018, 2020c). USVs were presented to individual subjects using an established playback paradigm (Wöhr et al., 2016; Berg et al., 2018), including the USV stimulus previously demonstrated to elicit social approach (behavior shown in Berg et al., 2020c). The USV stimulus consisted of 221 natural 50 kHz USVs recorded from a naive male rat during exploration of a cage containing a recently separated cage mate. A 3.5 s sequence of 13 calls was repeated 17 times such that 221 50 kHz calls were presented within 1 min. Response vocalizations were recorded with an overhead ultrasonic microphone (Avisoft Bioacoustics), and the number of calls emitted during the minute of playback were counted by a trained observer blinded to genotype and classified as high (50 kHz) or low (short 22 kHz) frequency using a threshold of 33 kHz.

### Juvenile social play

At PND 38 ± 1, social play behavior was assessed following a protocol described previously (Berg et al., 2018, 2020a,b). Each subject rat was placed with a freely moving, unfamiliar, strain-, sex-, and age-matched WT stimulus rat for 10 min in a clean, empty test arena (illuminated to ∼30 lux) containing a thin layer of clean bedding. In order to facilitate social play, each subject and stimulus animal was socially isolated in a separate holding room for 30 min before the test. Stimulus animals were generated from WT Sprague Dawley breeders (Envigo) and handled in a standardized manner (5 min on 3 d) before the assay. The interaction was video-recorded, and behaviors were later scored by a trained observer blinded to genotype as follows: Social sniffing: sniffing the stimulus rat's face, body, or tail; Anogenital sniffing: sniffing the stimulus rat's anogenital region; Self-grooming: subject grooming itself; Exploring: sitting, walking, rearing, or sniffing the ground or wall; Following or chasing: following (walking pace) or chasing (running pace) the stimulus rat; Rough-and-tumble playing: accelerated movement involving chasing, pouncing, pinning, tumbling, and/or boxing which requires the stimulus rat's participation (i.e., reciprocity); Push past: directed movement toward the stimulus rat to get next to, or move closely past, without sniffing or otherwise engaging; Push under or crawl over: head dip under the stimulus rat's belly or completely stepping over the stimulus rat; and Pounce: both paws placed via leap or directed movement onto the stimulus rat's back. Blind scoring was possible since *Ube3a*^mat–/pat+^ rats have normal body weight and are physically indistinguishable from their WT littermates (Berg et al., 2020c).

### Olfactory discrimination

At PND 42 ± 3, the ability of rats to discriminate between a social and nonsocial odor was tested by measuring the time spent investigating odor-saturated cotton swabs. Subjects were individually tested in clean chambers (40.6 cm [length] × 40.6 cm [width] × 28 cm [height]) dimly illuminated to ∼30 lux. On the day before the test, rats were habituated to the test chamber containing a clean dry cotton swab (15.2 cm l) for 20 min. The tip of the swab was secured 3 cm above the floor in the center of the arena by being attached to the top of a clean weighted glass dome (7.6 cm [diameter] × 10 cm [height]) and angled downward. On the day of the test, rats were again habituated to the arena containing a clean dry cotton swab for 10 min, followed by a swab soaked in water, then vanilla (1:100 dilution; McCormick), and then a social scent. The social scent was collected by wiping a cotton swab in a zig zag pattern along the bottom of a cage of same sex but unfamiliar Sprague Dawley rats (Envigo). Each saturated swab was presented for 2 min, and the order of odor presentation was consistent across all animals. Time spent sniffing the swab soaked with vanilla scent and the swab soaked with social scent (defined as the nose within 2 cm of the cotton swab tip) was measured using videotracking software (EthoVision XT, Noldus Information Technology), which was subsequently validated manually.

### Juvenile gait

At PND 25, gait metrics were collected using the DigiGait automated treadmill system and analysis software (Mouse Specifics). Subjects were placed individually into the enclosed treadmill chamber and allowed to acclimate before the belt was turned on. The belt speed was slowly increased to a constant speed of 20 cm/s, at which each rat was recorded making clearly visible consecutive strides for 3-6 s.

### Juvenile contextual and cued fear conditioning

At PND 43 ± 1, learning and memory were assessed using an automated fear conditioning chamber (Med Associates) following methods previously described (Copping et al., 2017; Adhikari et al., 2019; Berg et al., 2020b). On day 1, rats were trained via exposure to a series of three noise-shock (conditioned stimulus-unconditioned stimulus; CS-US; 80 dB white noise, 0.7 mA foot shock) pairings inside a sound-attenuated chamber with specific visual, tactile, and odor cues. On day 2, contextual memory was tested by placing each subject back inside the training environment (no noise or foot shock occurred). On day 3, cued memory was evaluated by placing subjects into a novel context with altered visual, tactile, and odor cues. Following a period of exploration, the white noise CS was presented for 3 min. Time spent freezing was measured using VideoFreeze software (Med Associates).

### Prepulse inhibition of an acoustic startle response

At 9-10 weeks of age, prepulse inhibition was measured using a SR-Lab System (San Diego Instruments). Subjects were placed in a clear plastic cylinder, which was mounted onto a platform connected to piezoelectric transducers inside a sound-attenuating chamber with internal speakers. The background noise level in the chamber was 70 dB white noise. Each session consisted of a 5 min acclimation period followed by a pseudo-randomized presentation of 50 trials of five different trial types: one trial type was a 40 ms 120 dB startle stimulus, three trial types involved an acoustic prepulse (74, 82, or 90 dB) presented 120 ms before the 120 dB startle stimulus, and there were also trials with no startle stimulus to measure baseline movement inside the cylinder. Each trial type was presented in 10 blocks and was randomized within blocks. The intertrial interval varied randomly between 10 and 20 s. Percent PPI was calculated using the equation: % PPI = [1 – (Prepulse/Max Startle)] × 100.

### Spontaneous alternation

At 10 weeks of age, spontaneous alternation was measured by allowing rats to freely explore a novel Y-maze (black, opaque; arms: 53.3 cm [length] × 11.4 cm [width] × 27.9 cm [height]; illuminated to ∼30 lux) for 8 min. An overhead camera connected to videotracking software (EthoVision XT; Noldus Information Technology) was used to quantify the number of arm entries, the number of errors (defined as the sum of direct and indirect revisits to an arm), the number of spontaneous alternations (defined as consecutively visiting all three arms without any revisit), and the maximum number of possible alternations for the entire session.

### LTP

#### Acute slice preparation

At 12-13 weeks of age, subjects were deeply anesthetized with isoflurane; and following decapitation, the brain was rapidly removed and submerged in ice-cold, oxygenated (95% O_2_/5% CO_2_) ACSF containing the following (in mm): 124 NaCl, 4 KCl, 25 NaHCO_3_, 1 NaH_2_PO_4_, 2 CaCl_2_, 1.2 MgSO_4_, and 10 glucose. On an ice-cold plate, the brain hemispheres were separated, blocked, and the hippocampi removed. The 400-μm-thick slices were then cut using a McIlwain tissue chopper (Brinkman). Slices from the dorsal third of the hippocampus were used. Slices were incubated at 33°C for 20 min and then maintained in submerged-type chambers that were continuously perfused (2-3 ml/min) with ACSF and allowed to recover for at least 1.5-2 h before recordings. Just before start of experiments, slices were transferred to a submersion chamber on an upright Olympus microscope, perfused with 30.4°C normal ACSF saturated with 95% O_2_/5% CO_2_.

#### Electrophysiological recordings

A bipolar, nichrome wire stimulating electrode (MicroProbes) was placed in stratum radiatum of the CA1 region and used to activate Schaffer collateral/commissural fiber synapses. Evoked fEPSPs (basal stimulation rate = 0.033 Hz) were recorded in stratum radiatum using borosilicate pipettes (Sutter Instruments) filled with ACSF (resistance 5-10 mΩ). Submerged-type recording chambers were used for all recordings. All recordings were obtained with a MultiClamp 700B amplifier (Molecular Devices), filtered at 2 kHz, and digitized at 10 Hz. To determine response parameters of excitatory synapses, basal synaptic strength was quantified by comparing the amplitudes of presynaptic fiber volleys and postsynaptic fEPSP slopes for responses elicited by different intensities of SC fiber stimulation. Presynaptic neurotransmitter release probability was compared by paired-pulse facilitation experiments, performed at 25, 50, 100, and 250 ms stimulation intervals. LTP was induced by high-frequency stimulation (HFS) using a 2× tetanus (1-s-long train of 100 Hz stimulation) with a 10 s intertetanus interval. At the start of each experiment, the maximal fEPSP amplitude was determined and the intensity of presynaptic fiber stimulation was adjusted to evoke fEPSPs with an amplitude ∼40%-50% of the maximal amplitude. The average slope of EPSPs elicited 55-60 min after HFS (normalized to baseline) was used for statistical comparisons.

### MRI

At 6.5 months of age, *ex vivo* neuroimaging was conducted by following a protocol previously described (Berg et al., 2018, 2020c). Brains were flushed and fixed via transcardial perfusion with 50 ml PBS containing 10 U/ml heparin and 2 mm ProHance (gadolinium contrast agent; Bracco Diagnostics) followed by 50 ml 4% PFA in PBS containing 2 mm ProHance. Brains were incubated in the 4% PFA solution at 4°C for 24 h, transferred to a 0.02% sodium azide PBS solution, and then incubated at 4°C for at least 1 month before being scanned. MRI of the brains within their skulls was conducted using a multichannel 7.0 Tesla scanner (Agilent Technologies). Seven custom millipede coils were used to image the brains in parallel (Bock et al., 2005; Lerch et al., 2011). Parameters used in the anatomic MRI scans are as follows: T2-weighted 3D fast spin echo sequence, with a cylindrical acquisition of k-space, and with a TR of 350 ms, and TEs of 10.5 ms per echo for 12 echoes, FOV 36 × 36 × 40 mm^3^, and a matrix size of 456 × 456 × 504, giving an image with 0.079 mm isotropic voxels (Spencer Noakes et al., 2017). The current scan time for this sequence is ∼ 3 h.

To visualize and compare any changes in the rat brains, the images were linearly and nonlinearly registered together using the pydpiper framework. Registrations were performed using a combination of mni_autoreg tools (Collins et al., 1994) and ANTS (advanced normalization tools) (Avants et al., 2011). Following registration, a population atlas was created representing the average anatomy of the study sample. At the end of the registration process, all the scans were deformed into alignment with one another in an unbiased fashion. This allows for analysis of the deformations required to register the brains together, which can be used to assess the volume of the individual brains and compared them to one another (Bishop et al., 2006; Lerch et al., 2008a,b,c; Nieman et al., 2010, 2018). For comparisons to the juvenile brains, a separate registration pipeline was used that included all the brains from this study as well as the previous Berg et al. (2020c) study. Volumetric differences were calculated on a regional and a voxelwise basis. An in-house manually segmented hierarchical rat brain atlas was used to calculate the volumes of 52 different segmented structures. These structures were derived from multiple atlases (Dorr et al., 2008; Steadman et al., 2014) and then modified for use in the rat brain.

### Experimental design and statistical analyses

Statistical analyses were performed using GraphPad Prism 8 statistical software (GraphPad Software). Clampex 10.6 software suite (Molecular Devices) was used for analyzing electrophysiological data. Congruent with previous studies, no significant sex differences were detected, so the results herein include both males and females. Effect sizes and power were determined using Cohen's *d*.

#### Analysis of behavior and LTP

For single comparisons between two groups, either a Student's *t* test or Mann–Whitney *U* test was used. Data that passed distribution normality tests, were collected using continuous variables, and had similar variances across groups were analyzed via Student's *t* test. Alternatively, a Mann–Whitney *U* test was used. Either a two-way ANOVA or two-way repeated-measures ANOVA was used to analyze the effects of genotype and a second factor. In repeated-measures ANOVA, genotype was the between-group factor and time, limb set, test phase, scent, or prepulse intensity was the within-group factor. *Post hoc* comparisons were performed following a significant main effect or interaction and were conducted using Holm–Sidak's multiple comparisons test controlling for multiple comparisons. Data points within 2 SDs of the mean were included, all significance tests were two-tailed, and a *p* value of < 0.05 was considered significant.

#### Analysis of MRI

Statistical analyses were used to compare both the absolute and relative volumes voxelwise as well as across the 52 different hierarchical structures in the rat brains. Absolute volume was calculated as mm^3^, and relative volume was assessed as a measure of % total brain volume. Voxelwise and regional differences were assessed using linear models. All image analysis tools and software are available on Github (https://github.com/Mouse-Imaging-Centre). Multiple comparisons were controlled for using the false discovery rate (Genovese et al., 2002).

## Results

### Overabundant emission of laughter-like 50 kHz calls in juvenile *Ube3a*^mat–/pat+^ rats

**Figure 1.**
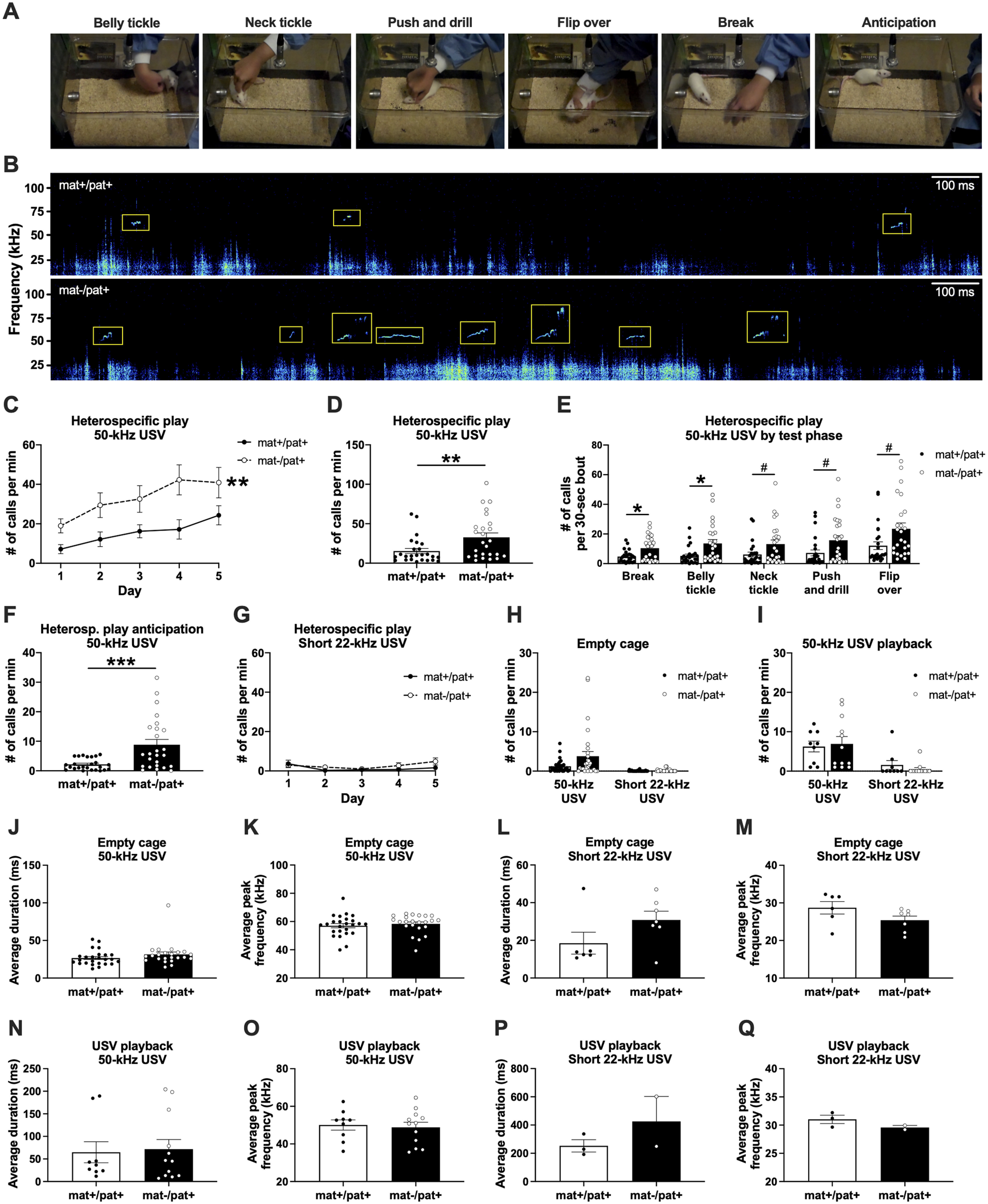
Overabundant emission of laughter-like 50 kHz calls in juvenile *Ube3a*^mat–/pat+^ rats. ***A***, Example images of the manipulations used to mimic social play and elicit USVs. ***B***, Example spectrograms of USVs from a WT littermate control (*Ube3a*^mat+/pat+^; mat+/pat+; top) and *Ube3a*^mat–/pat+^ rat (mat–/pat+; bottom). ***C***, Across 5 d of heterospecific play sessions, 50 kHz USV emission increased with repeated testing in both mat–/pat+ (*n* = 25) and controls (*n* = 25), but the emission rate was substantially elevated in mat–/pat+. ***D***, On average, mat–/pat+ rats produced 50 kHz USVs at more than twice the rate of controls. ***E***, Specifically, 50 kHz calling was abnormally high during the break and belly tickle phases, with trending increases during neck tickle, push and drill, and flip over. ***F***, Before the onset of play, mat–/pat+ rats emitted anticipatory 50 kHz USVs at >3 times the rate of controls. ***G***, Production of short 22 kHz USVs was low, did not differ between genotypes, and did not change over subsequent play sessions. ***H***, The rates of 50 kHz and short 22 kHz calling during empty cage exploration were comparable between genotypes (mat+/pat+, *n* = 32; mat–/pat+, *n* = 29), as were the (***I***) 50 kHz and short 22 kHz calling rates in response to hearing playback of conspecific 50 kHz USV (mat+/pat+, *n* = 9; mat–/pat+, *n* = 12). Call features did not differ by genotype: ***J***, The average duration and (***K***) peak frequency of spontaneous 50 kHz calls made during exploration of an empty cage were comparable between mat–/pat+ (*n* = 23) and mat+/pat+ rats (*n* = 25). ***L***, The average duration and (***M***) average peak frequency of short 22 kHz calls made within an empty cage were also similar between genotypes (mat+/pat+, *n* = 6; mat–/pat+, *n* = 7). ***N***, For 50 kHz USVs emitted in response to hearing playback of natural prerecorded 50 kHz rat USVs, average duration and (***O***) average peak frequency were comparable between mat–/pat+ rats (*n* = 12) and WT littermates (*n* = 9). ***P***, There was no genotype effect on the average duration or (***Q***) average peak frequency of short 22 kHz calls made during USV playback (mat+/pat+, *n* = 3; mat–/pat+, *n* = 2). Of note, long 22 kHz USVs known to function as “alarm calls” were very rarely observed, indicating that our paradigms were not aversive. Data are mean ± SEM. ***C***, **p < 0.01, repeated-measures ANOVA. ***D***, ***F***, ***p < 0.001, **p < 0.01, Mann–Whitney *U* test. ***E***, *p < 0.05, ^#^*p* < 0.06, repeated-measures ANOVA, Holm-Sidak's *post hoc*.

Since abnormal expressive communication and elevated rates of positive affect are key clinical features of AS, we sought to quantify these characteristics in *Ube3a*^mat–/pat+^ and *Ube3a*^mat+/pat+^ (WT) rats. While vocalizations are readily collected during social play, recording USVs from multiple interacting animals makes it difficult to determine which animal made each call. We therefore took advantage of the fact that rats emit laughter-like 50 kHz calls when social play is simulated by an experimenter via tickling and other physical maneuverings (Burgdorf and Panksepp, 2001; Burgdorf et al., 2005, 2008; Ishiyama and Brecht, 2016). We implemented a standardized heterospecific play procedure (Fig. 1*A*) to elicit USVs (Fig. 1*B*) while maintaining full confidence in the identity of the caller and controlling for the level of physical interaction across subjects.

We discovered that, while both groups increased 50 kHz USV emission across consecutive sessions, *Ube3a*^mat–/pat+^ emitted a substantially elevated level of 50 kHz USVs (Fig. 1*C*; *F*_Genotype(G)_(1,48) = 7.351, *p* = 0.009; *F*_Day(D)_(3.007,144.3) = 10.82, *p* < 0.0001; *F*_D×G_(4192) = 1.052, *p* > 0.05). In total, *Ube3a*^mat–/pat+^ emitted an average of 33 ± 5 USVs per minute (mean ± SEM), more than twice the rate of controls, which produced an average of 15 ± 3 calls per minute (Fig. 1*D*; *U* = 175, *p* = 0.007, *d* = 0.77). A closer examination revealed that 50 kHz USVs were elevated during the break and belly tickle phases (Fig. 1*E*; *F*_G_(1,48) = 6.927, *p* = 0.011; *F*_Phase (P)_(1.722,82.64) = 27.83, *p* < 0.0001; *F*_P×G_(4192) = 2.075, *p* > 0.05; *post hoc*: break, *p* = 0.023, *d* = 0.85; belly tickle, *p* = 0.023, *d* = 0.84), although calling during the other phases also trended higher, providing strong evidence of elevated positive affect and a high hedonic impact of the assay (neck tickle, *p* = 0.057, *d* = 0.62; push and drill, *p* = 0.057, *d* = 0.65; flip over, *p* = 0.057, *d* = 0.69). There was no effect of sex, nor an interaction with sex (*p* > 0.05), for any parameter.

Additionally, 50 kHz USVs were more frequently emitted during the anticipation period immediately before the play sessions (Fig. 1*F*; *U* = 146.5, *p* = 0.001, *d* = 1.04). In total, across all four anticipation time points (days 2-5), *Ube3a*^mat–/pat+^ emitted an average of 9 ± 2 USVs per minute (mean ± SEM), >4 times the rate of WTs, which produced an average of 2 ± 0.4 calls per minute. This indicates that *Ube3a*^mat–/pat+^ predicted the impending onset of play and that the interaction had a high degree of incentive salience.

Excessive vocalization by *Ube3a*^mat–/pat+^ rats was specific to 50 kHz USVs. Production of short 22 kHz USV, which are emitted in modest amounts during play, was low and did not differ between genotypes (Fig. 1*G*; *F*_G_(1,48) = 1.771, *p* > 0.05; *F*_D_(1.825,87.62) = 3.160, *p* > 0.05; *F*_D×G_(4192) = 1.330, *p* > 0.05). Elevated 50 kHz calling by *Ube3a*^mat–/pat+^ was also specific to being provoked by heterospecific play, as 50 kHz and short 22 kHz USV production was normal during exploration of an empty cage (albeit a slight trend toward more 50 kHz USV; Fig. 1*H*; 50 kHz, *U* = 374.5, *p* > 0.05; 22 kHz, *U* = 433, *p* > 0.05, *d* = 0.56) and in response to the acoustic presentation of 50 kHz USVs (Fig. 1*I*; 50 kHz, *U* = 53, *p* > 0.05; 22 kHz, *U* = 44.50, *p* > 0.05). No gross abnormalities in call structure were observed. Specifically, 50 kHz calls were of normal duration and peak frequency (Fig. 1*J*: *U* = 205, *p* > 0.05; Fig. 1*K*: *U* = 240, *p* > 0.05; Fig. 1*N*: *U* = 52, *p* > 0.05; Fig. 1*O*: *t*_(19)_ = 0.3179, *p* > 0.05), suggesting that increased heterospecific play 50 kHz call numbers were not inflated by shorter or broken calls. Duration and peak frequency of 22 kHz USVs were also comparable between genotypes (Fig. 1*L*: *U* = 12, *p* > 0.05; Fig. 1*M*: *t*_(11)_ = 1.699, *p* > 0.05; Fig. 1*P*: *U* = 1, *p* > 0.05; Fig. 1*Q*: *U* = 1, *p* > 0.05). Since the average duration of the juvenile 22 kHz USV fell short of the usual durations of adult “typical 22 kHz” USVs, we herein refer to them as “short 22 kHz” USVs.

### Intact social interest but deficient expression of key social interaction behaviors in juvenile *Ube3a*^mat–/pat+^ rats

**Figure 2.**
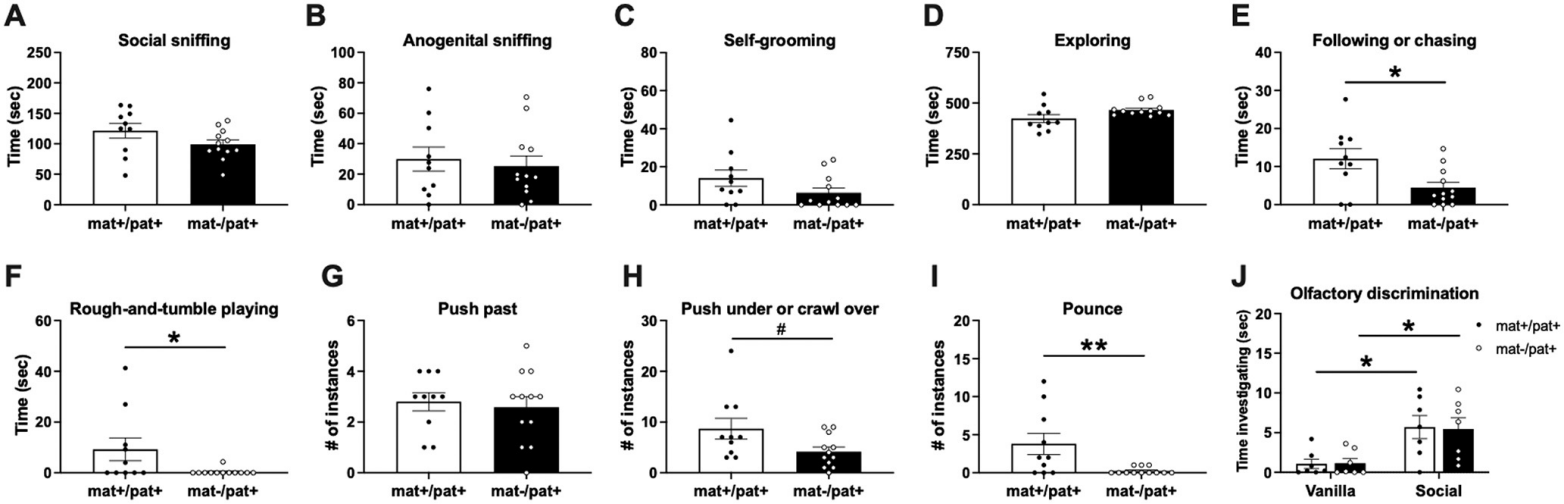
Intact social interest but deficient expression of key social interaction behaviors in juvenile *Ube3a*^mat–/pat+^ rats. ***A***, During a 10 min interaction session with a novel same-sex WT conspecific, *Ube3a*^mat–/pat+^ rats (mat–/pat+; *n* = 12) spent similar amounts of time social sniffing, (***B***) anogenital sniffing, (***C***) self-grooming, and (***D***) exploring the arena compared with WT littermate controls (Ube3a^mat+/pat+^; mat+/pat+; *n* = 10). ***E***, Robust deficits, however, were discovered in the time spent following or chasing and (***F***) rough-and-tumble playing. ***G***, The number of push pasts were similar across genotypes, but (***H***) there was a trend for mat–/pat+ to less frequently push under or crawl over and (***I***) mat–/pat+ rats did not perform nearly as many pounces as WT littermates. ***J***, A separate test of olfactory discrimination revealed normal sniff times of social and nonsocial scents. Time spent investigating novel odors was similar for mat–/pat+ (*n* = 7) and mat+/pat+ rats (*n* = 7) and both groups spent more time investigating a social scent compared with a non-social vanilla odor. Data are mean ± SEM. ***E–I***, **p < 0.01, *p < 0.05, ^#^*p* < 0.065, Mann–Whitney *U* test. ***J***, *p < 0.05, repeated-measures ANOVA, Holm-Sidak's *post hoc*.

We sought to investigate whether elevated 50 kHz USV emission in *Ube3a*^mat–/pat+^ rats was associated with greater social engagement with a conspecific. Starting at ∼2 weeks of age, rats play fight with each other by chasing, pouncing, pinning, and wrestling in a manner similar to cats and dogs. Through developmental experience, they learn how to appropriately initiate, engage in, and terminate play bouts with others. In order to more closely examine social behavior and the nuanced reciprocal interactions of social play, we gave juvenile subjects the opportunity to freely interact with a conspecific (Meaney and Stewart, 1981; Panksepp, 1981; Pellis and Pellis, 1998, 2017). Despite emission of more 50 kHz calling during heterospecific play, *Ube3a*^mat–/pat+^ rats showed a normal degree of interest in the stimulus animal, demonstrated by the amounts of time spent social sniffing (Fig. 2*A*; *t*_(20)_ = 1.646, *p* > 0.05) and anogenital sniffing (Fig. 2*B*; *t*_(20)_ = 0.4457, *p* > 0.05). Putting forth a similar level of investigative effort suggested that *Ube3a*^mat–/pat+^ are just as motivated for social interaction as controls. Levels of self-grooming (Fig. 2*C*; *U* = 38, *p* > 0.05) and arena exploration (Fig. 2*D*; *U* = 30, *p* > 0.05) were also normal, but *Ube3a*^mat–/pat+^ spent markedly less time following or chasing the stimulus rat (Fig. 2*E*; *U* = 29, *p* = 0.041, *d* = 1.11). The key observation was the reduced time spent rough-and-tumble playing (Fig. 2*F*; *U* = 33.50, *p* = 0.029, *d* = 0.89) compared with WTs. In an attempt to reconcile the near lack of play with intact levels of social interest, we quantified specific components of rough-and-tumble play. While the number of side-to-side social contacts via push pasts were similar across genotypes (Fig. 2*G*; *t*_(20)_ = 0.3852, *p* > 0.05), there was a trending reduction in the number of push under or crawl overs (Fig. 2*H*; *U* = 31.5, *p* = 0.061, *d* = 0.89) and almost a complete lack of pouncing in *Ube3a*^mat–/pat+^ (Fig. 2*I*; *U* = 24, *p* = 0.008, *d* = 1.14). A separate test of olfaction was used to rule out an olfactory deficit as a confounder of social investigation (Fig. 2*J*; *F*_Genotype(G)_(1,12) = 0.0066, *p* = 0.937; *F*_Scent(S)_(1,12) = 14.20, *p* = 0.003; *F*_S×G_(1,12) = 0.0165, *p* = 0.900; *post hoc*: mat+/pat+, *p* = 0.035; mat–/pat+, *p* = 0.035).

### Abnormal gait in *Ube3a*^mat–/pat+^ rats

**Figure 3.**
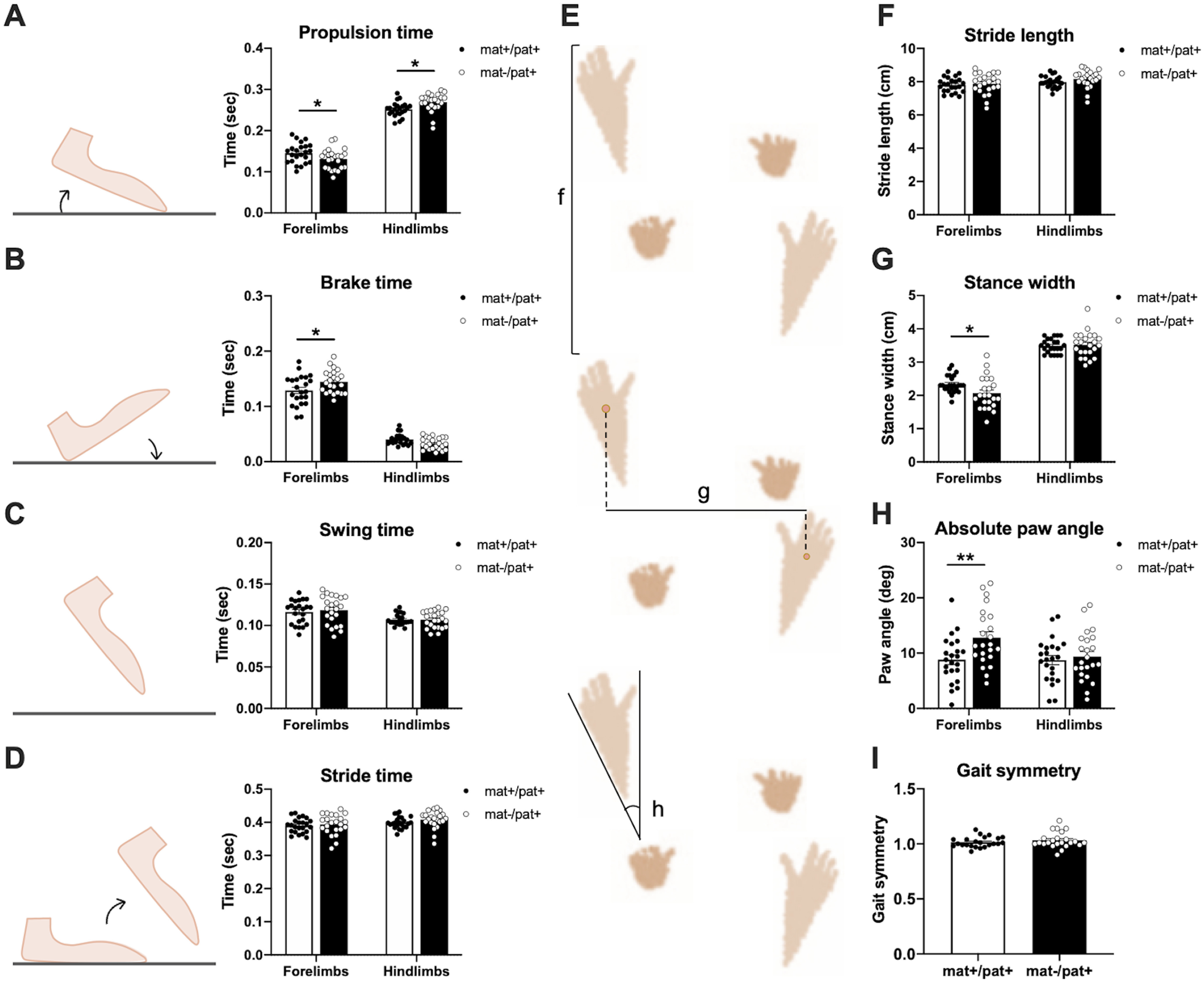
Abnormal gait in *Ube3a*^mat–/pat+^ rats. ***A***, While treadmill walking, *Ube3a*^mat–/pat+^ rats (mat–/pat+; *n* = 23) displayed aberrant propulsion time (time from maximal paw contact with belt to just before liftoff) in both sets of limbs. Compared with WT littermates (*Ube3a*^mat+/pat+^; mat+/pat+; *n* = 23), propulsion time was decreased in the forelimbs and increased in hindlimbs. ***B***, Brake time (time from initial to maximal paw contact with belt) was significantly elevated in the forelimbs of mat–/pat+ while a trending reduction in hindlimb brake time was found (*p* = 0.150). ***C***, Swing time (no paw contact with the belt) and (***D***) stride time (sum of swing and stance time) were similar across genotypes. ***E***, Example paw prints illustrating the spatial gait parameters depicted in ***F–H***. ***F***, Stride length did not differ between groups, but (***G***) forelimb stance width was narrower and (***H***) absolute paw angle for the forelimbs was greater, indicating more external rotation in mat–/pat+ rats. ***I***, No significant difference in gait symmetry (ratio of forelimb to hindlimb stepping frequency) was detected. Data are mean ± SEM. **p < 0.01, *p < 0.05, repeated-measures ANOVA, Holm-Sidak's *post hoc*.

In an effort to assess the potential contribution of motor defects to social play behavior, we explored motor dysfunction, which is a core clinical feature of AS prevalent in mouse models (Huang et al., 2013; Leach and Crawley, 2018) and hypothesized by our group to underlie the open field, rotarod, and marble-burying phenotypes of AS mouse models. Previously, we discovered lower open field vertical activity in *Ube3a*^mat–/pat+^ rats, whereas other activity indices were typical (Berg et al., 2020c). Using the DigiGait automated treadmill system, we found that juvenile *Ube3a*^mat–/pat+^ rats displayed robust abnormalities in limb propulsion time, indicating reduced limb strength and less force produced per unit time compared with WTs (Fig. 3*A*; *F*_Genotype(G)_ (1,44) = 0.0684, *p* > 0.05; *F*_Limbs(L)_(1,44) = 776.8, *p* < 0.0001; *F*_L×G_(1,44) = 12.80, *p* < 0.001; *post hoc*: forelimbs, *p* = 0.030, *d* = 0.60; hindlimbs, *p* = 0.022, *d* = 0.85; Fig. 3*B*; *F*_G_(1,44) = 1.012, *p* > 0.05, *F*_L_(1,44) = 687.0, *p* < 0.0001; *F*_L×G_(1,44) = 9.391, *p* = 0.004; *post hoc*: forelimbs, *p* = 0.010, *d* = 0.65). No abnormalities in swing time (Fig. 3*C*; *F*_G_(1,44) = 0.1209, *p* > 0.05, *F*_L_(1,44) = 22.62, *p* < 0.0001; *F*_L×G_(1,44) = 0.2552, *p* > 0.05) or total stride time (Fig. 3*D*; *F*_G_(1,44) = 0.9166, *p* > 0.05; *F*_L_(1,44) = 13.24, *p* < 0.001; *F*_L×G_(1,44) = 0.7566, *p* > 0.05) were discovered, suggesting that the opposing effects of propulsion and brake time canceled each other out. Stride length was normal, which was surprising given the published Zeno Walkway data in humans (Grieco et al., 2018), but lends to the hypothesis that *Ube3a*^mat–/pat+^ have limb weakness since more time was required to produce force for an equal length step (Fig. 3*F*; *F*_G_(1,44) = 0.9460, *p* > 0.05; *F*_L_(1,44) = 12.70, *p* < 0.001; *F*_L×G_(1,44) = 0.7719, *p* > 0.05). Forelimb stance width was reduced (Fig. 3*G*; *F*_G_(1,44) = 1.605, *p* > 0.05; *F*_L_(1,44) = 939.0, *p* < 0.0001; *F*_L×G_(1,44) = 12.46; *post hoc*: forelimbs, *p* = 0.022, *d* = 0.69) while an elevated forelimb paw angle indicated greater degree of external rotation and splaying (Fig. 3*H*; *F*_G_(1,44) = 5.957, *p* = 0.019; *F*_L_(1,44) = 3.726, *p* > 0.05; *F*_L×G_(1,44) = 3.497, *p* > 0.05; *post hoc*: forelimbs, *p* = 0.006, *d* = 0.86), which has been associated with ataxia, spinal cord injury, and demyelinating disease (Powell et al., 1999). The observed effects were not attributable to differences in body length (data not shown; *U* = 244.5, *p* > 0.05) or body width (data not shown; *t*_(44)_ = 0.2719, *p* > 0.05); and despite abnormalities in some temporal and postural components of gait, the coordination metric of gait symmetry was unaltered (Fig. 3*I*; *t*_(44)_ = 1.023, *p* > 0.05).

### Impaired learning and memory in *Ube3a*^mat–/pat+^ rats

**Figure 4.**
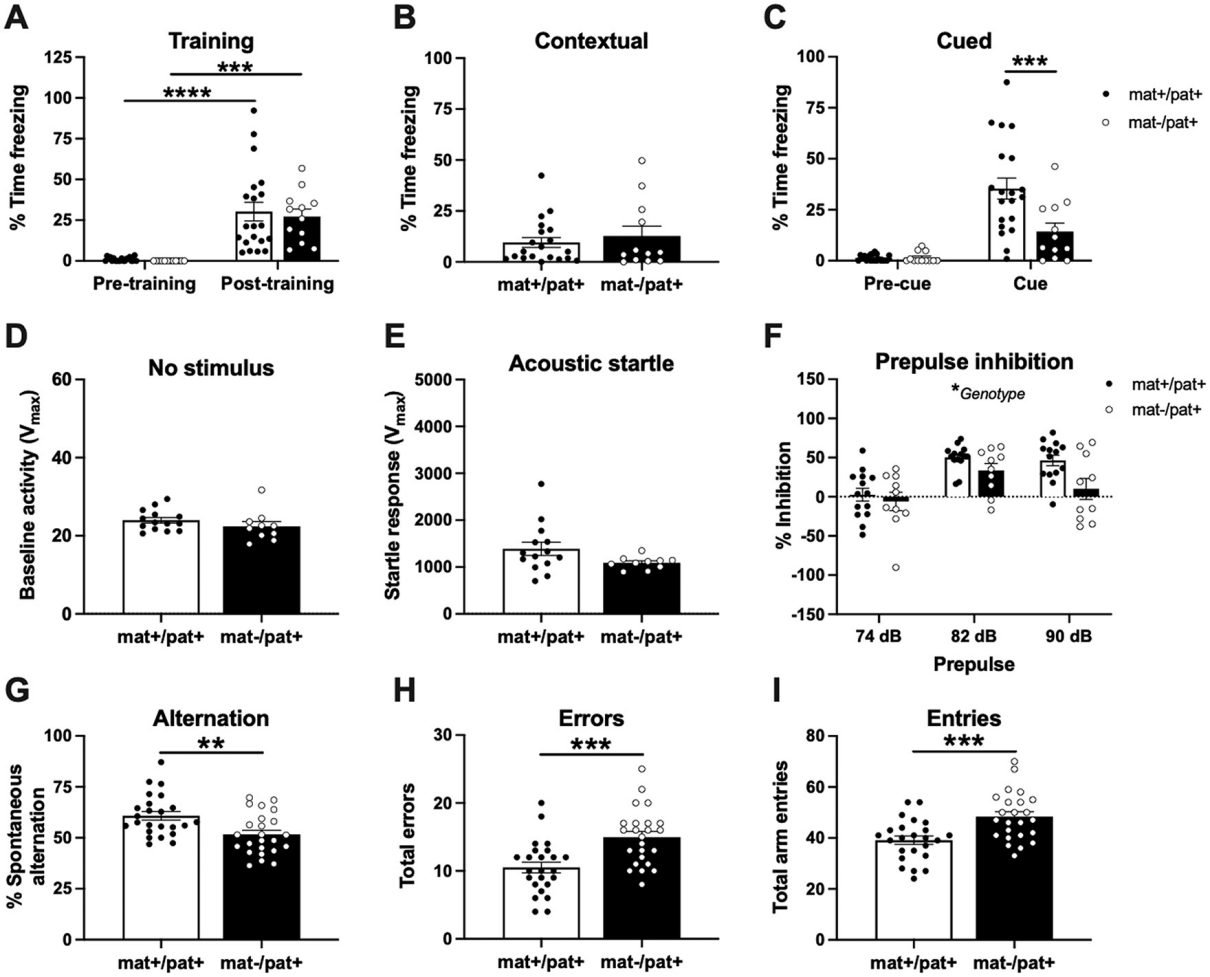
Impaired learning and memory in *Ube3a*^mat–/pat+^ rats. ***A***, During fear conditioning training, juvenile *Ube3a*^mat–/pat+^ (mat–/pat+; *n* = 12) and WT littermate controls (*Ube3a*^mat+/pat+^; mat+/pat+; *n* = 20) showed similar increases in freezing after training. ***B***, When returned to the training context 24 h following training, mat–/pat+ rats exhibited a similar level of freezing to WTs. ***C***, When introduced to a novel context 48 h after training, no difference in freezing pre-cue was found, but mat–/pat+ rats froze for less than half the time of WTs during presentation of the auditory cue. A separate sensorimotor test confirmed intact auditory sensitivity: ***D***, Baseline activity within the testing apparatus was comparable between mat–/pat+ (*n* = 10) and mat+/pat+ rats (*n* = 14). ***E***, There was no effect of genotype on the startle response to a 120 decibel (dB) startle stimulus. ***F***, Prepulse inhibition of the startle response was generally reduced in adult mat–/pat+ rats. ***G***, Spontaneous arm alternation during Y-maze exploration was significantly reduced in adult mat–/pat+ rats (*n* = 24) compared with WT littermates (*n* = 24). ***H***, Mat–/pat+ rats made 40% more errors and (***I***) made more entries into the maze arms. Error bars indicate mean ± SEM. ***A***, ***C***, ****p < 0.0001, ***p < 0.001, repeated-measures ANOVA, Holm-Sidak's *post hoc*. ***F***, *p < 0.05, repeated-measures ANOVA main effect. ***G–I***, ***p < 0.001, **p < 0.01, Student's *t* test.

Learning and memory impairments, which are characteristic of AS, may hinder the ability of *Ube3a*^mat–/pat+^ rats to learn via developmental experience how to appropriately engage in social interactions. We therefore probed for a juvenile learning and memory deficit using a fear conditioning assay previously used to detect a deficit in adulthood (Dodge et al., 2020). Following successful fear conditioning (Fig. 4*A*; *F*_Phase(P)_(1,30) = 48.47, *p* < 0.0001; *F*_Genotype(G)_(1,30) = 0.2203, *p* > 0.05; *F*_P×G_(1,30) = 0.0613, *p* > 0.05; *post hoc*: mat+/pat+, *p* < 0.0001; mat–/pat+, *p* < 0.001), juvenile *Ube3a*^mat–/pat+^ displayed normal levels of freezing in response to the training context (Fig. 4*B*; *U* = 117.5, *p* > 0.05) but a robust deficit in cued fear memory 48 h after training (Fig. 4*C*; *F*_G_(1,30) = 7.395, *p* = 0.011; *F*_P_(1,30) = 42.36, *p* < 0.0001; *F*_P×G_(1,30) = 8.699, *p* = 0.006; *post hoc*: pre-cue, *p* > 0.05; cue, *p* < 0.001, *d* = 1.10). We assessed the potentially confounding variable of impaired sensorimotor processing by measuring the startle response to an intense acoustic stimulus and quantifying the reduction in startle response following prepulses of varying intensities. Both baseline activity (Fig. 4*D*; *t*_(22)_ = 1.735, *p* > 0.05) and the acoustic startle response of *Ube3a*^mat–/pat+^ rats were normal (Fig. 4*E*; *t*_(22)_ = 1.157, *p* > 0.05), indicating intact hearing abilities. While there was a significant main effect of genotype on prepulse inhibition, indicative of a sensorimotor gating deficit (*F*_Genotype(G)_(1,22) = 4.740, *p* =0.041, *d* = 0.88; *F*_Prepulse(P)_(1.898,41.75) = 20.64, *p* < 0.0001; *F*_P×G_(2,44) = 2.127, *p* = 0.1312), *post hoc* testing revealed no significant difference between groups at any individual prepulse level (Fig. 4*F*; 74 dB, *p* > 0.05, *d* = 0.25; 82 dB, *p* > 0.05, *d* = 0.73; 90 dB, *p* > 0.05, *d* = 1.05).

As an additional assessment of cognitive functioning, we quantified spontaneous alternation during exploration of a Y-maze and found that *Ube3a*^mat–/pat+^ rats displayed reduced spontaneous alternation compared with WTs (Fig. 4*G*; *t*_(46)_ = 3.115, *p* < 0.01, *d* = 0.90). *Ube3a*^mat–/pat+^ rats made 40% more errors (Fig. 4*H*; *t*_(46)_ = 3.827, *p* < 0.001, *d* = 1.10) and more arm entries (Fig. 4*I*; *t*_(46)_ = 3.620, *p* < 0.001, *d* = 1.04) despite no difference in the total distance moved (data not shown; Student's *t* test: *t*_(46)_ = 1.721, *p* > 0.05). Together, these metrics indicate additional cognitive deficits in the *Ube3a*^mat–/pat+^ rats that were not confounded by a locomotor deficiency.

### Reduced hippocampal LTP in *Ube3a*^mat–/pat+^ rats

**Figure 5.**
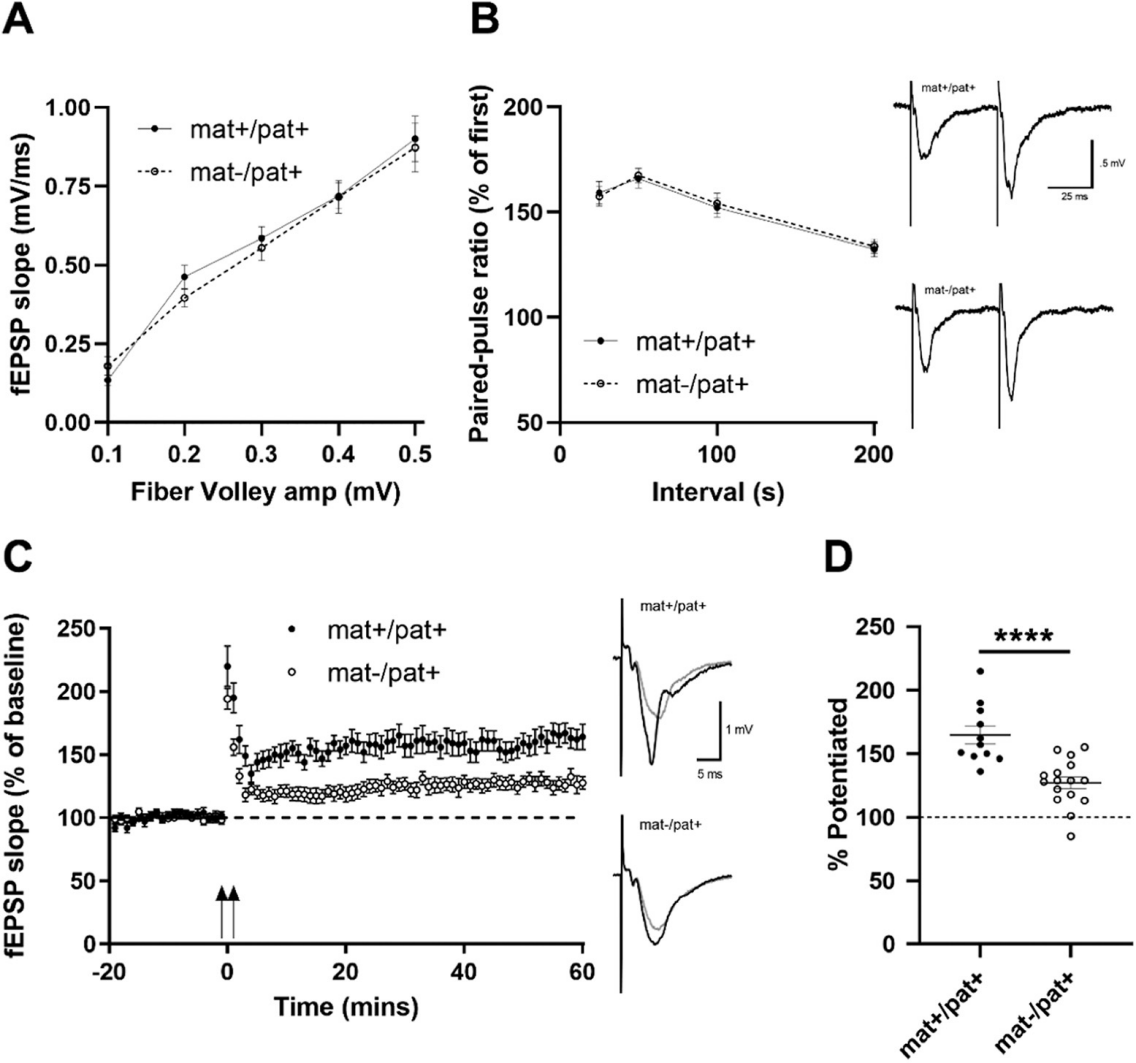
Reduced hippocampal LTP in *Ube3a*^mat–/pat+^ rats. ***A***, Normal basal synaptic transmission as measured by presynaptic fiber volley amplitudes and postsynaptic fEPSP slopes for responses elicited by different intensities of SC fiber stimulation in *Ube3a*^mat–/pat+^ (mat–/pat+; *n* = 16) and WT littermate (*Ube3a*^mat+/pat+^; mat+/pat+; *n* = 11) hippocampal slices. ***B***, Paired-pulse facilitation was unchanged at mat–/pat+ SC-CA1 synapses compared with mat+/pat+ (*n* = 15 mat+/pat+ and *n* = 20 mat–/pat+ slices). Right, Traces represent fEPSPs evoked by stimulation pulses delivered with a 50 ms interpulse interval. Calibration: 0.5 mV, 25 ms. ***C***, HFS-induced LTP in mat+/pat+ (*n* = 11) was significantly greater compared with mat–/pat+ (*n* = 16). Right, Traces represent superimposed fEPSPs recorded during baseline and 60 min after HFS. Calibration: 1 mV, 5 ms. ***D***, Summary graph of average percentage potentiation relative to baseline demonstrating that mat+/pat+ exhibited significantly enhanced SC-CA1 LTP at 60 min after HFS (delivered at time = 0), fEPSPs were potentiated to 160 ± 7% of baseline in mat+/pat+ (*n* = 11) and were 127 ± 5% of baseline in mat–/pat+ slices (*n* = 16). Data were collected from 2 rats per genotype. ****p < 0.0001, Student's *t* test.

To elucidate the neurobiology underpinning the learning and memory deficits of *Ube3a*^mat–/pat+^ rats, we quantified LTP. Previous studies in mouse models of AS have shown that LTP, a major cellular mechanism underlying learning and memory (Collingridge and Isaac, 2003), is impaired (Jiang et al., 1998; van Woerden et al., 2007; Daily et al., 2011). Here, we examined hippocampal LTP in adult *Ube3a*^mat–/pat+^ rats compared with WT littermate controls. We found hippocampal-dependent contextual fear memory intact at the juvenile age, but a previous report detected a clear deficit in adults (Dodge et al., 2020), therefore we measured hippocampal LTP in adulthood. Basal synaptic strength (Fig. 5*A*; *F*_Genotype(G)_(1,92) = 0.2013, *p* > 0.05; *F*_Amplitude(A)_(5,111) = 94.04, *p* < 0.0001; *F*_G×A_(5,92) = 0.4107, *p* > 0.05) and paired-pulse ratio (Fig. 5*B*; *F*_G_(1,56) = 0.065, *p* > 0.05; *F*_Interval(I)_(3,76) = 20.96, *p* < 0.0001; *F*_G×I_(3,56) = 0.0758, *p* > 0.05) were unaltered in *Ube3a*^mat–/pat+^ rats, suggesting no change in baseline excitatory transmission. However, consistent with the mouse models of AS (Jiang et al., 1998; van Woerden et al., 2007; Daily et al., 2011), we found that the magnitude of LTP was reduced in *Ube3a*^mat–/pat+^ rats (Fig. 5*C*,*D*; *t*_(25)_ = 4.641, *p* < 0.0001, *d* = 1.78), suggesting a putative mechanism underlying impairment of learning and memory (Zucker, 1989; Jiang et al., 1998; Zucker and Regehr, 2002; van Woerden et al., 2007; Daily et al., 2011).

### Neuroanatomical pathology in *Ube3a*^mat–/pat+^ rats revealed by high-resolution MRI

**Figure 6.**
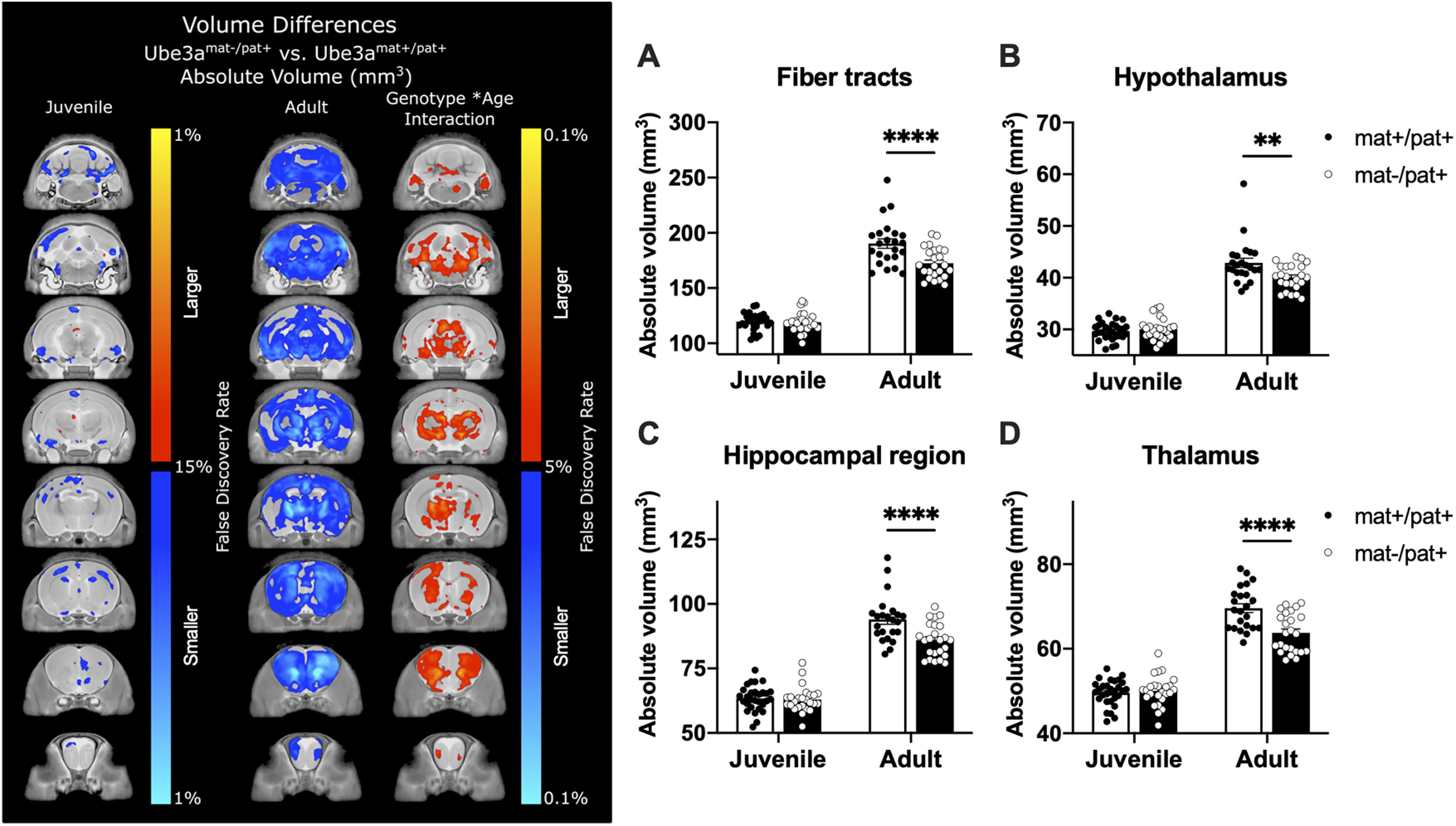
Neuroanatomical pathology in *Ube3a*^mat–/pat+^ rats revealed by high-resolution MRI. Left, Slice series comparing absolute volume (mm^3^) of juvenile and adult populations of *Ube3a*^mat–/pat+^ (mat–/pat+) rats and WT littermates (*Ube3a*^mat+/pat+^; mat+/pat+). Red to yellow represents increased volume compared with WT. Dark blue to light blue represents decreased volume. Leftmost column represents data on juvenile mat–/pat+ rats from Berg et al. (2020c). Middle column represents the same slices on the adult dataset presented here. Most notably, total brain volume was 6.0% smaller in mat–/pat+ rats compared with WT. Additionally, the third column represents the genotype × age interaction highlighting several ROIs (right), four of which are shown as follows: ***A***, fiber tracts; ***B***, the hypothalamus; ***C***, the hippocampal region; and ***D***, the thalamus. Full details of regional findings for adult animals and the interaction effect are described in Extended Data Figures 6-1 and 6-2. Group sizes: juvenile mat+/pat, *n* = 29; juvenile mat–/pat+, *n* = 25; adult mat+/pat+, *n* = 23; adult mat–/pat+, *n* = 24. Error bars indicate mean ± SEM. ****p < 0.0001, **p < 0.01, two-way ANOVA, Holm-Sidak's *post hoc*.

MRI revealed striking differences at 6.5 months of age in total brain volume, which was decreased by 6.0% in *Ube3a*^mat–/pat+^ rats (*q* = 0.04; Fig. 6; Extended Data Fig. 6-1). The overall brain volume difference was driven by decreases in the hippocampal region (−6.3%, *q* = 0.04), brainstem (−5.6%, *q* = 0.04), thalamus (−7.7%, *q* = 0.01), cerebellum (−9.0%, *q* = 0.02), and deep cerebellar nuclei (−12.3%, *q* = 0.0001). Additional differences were found throughout the white matter fiber tracts (−7.6%, *q* = 0.02), including but not limited to the cerebral peduncle (−7.6%, *q* = 0.02), internal capsule (−8.4%, *q* = 0.02), and arbor vita of the cerebellum (−11.7%, *q* = 0.0004). Moreover, trends were seen in other large white matter structures, including the corpus callosum (−6.7%, *q* = 0.06) and fornix system (−6.0%, *q* = 0.09). A complete list of the regional structural differences in both absolute (mm^3^) and relative (% total brain) volume is provided in Extended Data Fig. 6-1.

10.1523/JNEUROSCI.0925-21.2021.f6-1 Figure 6-1 Brain volumes for adult *Ube3a*^mat–/pat+^ rats and WT littermates. Download Figure 6-1, XLSX file.

10.1523/JNEUROSCI.0925-21.2021.f6-2 Figure 6-2 Age × genotype interaction for absolute brain volumes of juvenile and adult *Ube3a*^mat–/pat+^ rats and WT littermates. Download Figure 6-2, XLSX file.

As we had previously examined *Ube3a*^mat–/pat+^ rats at a juvenile age (PND 21) (Berg et al., 2020c), we felt an age × genotype comparison was warranted. Figure 6 highlights these changes in eight coronal slices, separately from both the previous work on juvenile rats and from the current data on adults. A combined dataset using both the juvenile and adult data were then used to examine a genotype × age interaction model, which revealed several regions to diverge with age and genotype: total brain volume (*q* = 0.048), caudoputamen (*q* = 0.03), white matter fiber tracts (*q* = 0.03; Fig. 6*A*; *F*_Age(A)_(1,97) = 546.5, *p* < 0.0001; *F*_Genotype(G)_(1,97) = 11.87, *p* < 0.001; *F*_A×G_(1,97) = 10.68, *p* = 0.002; *post hoc*: juvenile, *p* > 0.05; adult, *p* < 0.0001, *d* = 1.02), hypothalamus (*q* = 0.046; Fig. 6*B*; *F*_A_(1,97) = 460.2, *p* < 0.0001; *F*_G_(1,97) = 5.081, *p* = 0.026; *F*_A×G_(1,97) = 8.760, *p* = 0.004; *post hoc*: juvenile, *p* > 0.05; adult, *p* = 0.001, *d* = 0.82), hippocampal region (*q* = 0.046; Fig. 6*C*; *F*_A_(1,97) = 434.4, *p* < 0.0001; *F*_G_(1,97) = 10.89, *p* = 0.001; *F*_A×G_(1,97) = 8.760, *p* = 0.004; *post hoc*: juvenile, *p* > 0.05; adult, *p* < 0.0001, *d* = 1.02), and thalamus (*q* = 0.02; Fig. 6*D*; *F*_A_(1,97) = 430.2, *p* < 0.0001; *F*_G_(1,97) = 11.14, *p* = 0.001; *F*_A×G_(1,97) = 14.96, *p* < 0.001; *post hoc*: juvenile, *p* > 0.05; adult, *p* < 0.0001, *d* = 1.22). A full list of the regional genotype × age interactions is located in Extended Data Fig. 6-2. Voxelwise changes were also found throughout the brain of adult *Ube3a*^mat–/pat+^ rats compared with the juvenile age. The changes in the adults were substantially larger, signaling a more severe neuroanatomical phenotype with age (Fig. 6).

## Discussion

Indispensable to therapeutic development are *in vivo* studies using preclinical model systems. While mice have prevailed as the animal model of AS in recent decades (Jiang et al., 1998), the *Ube3a*^mat–/pat+^ rat offers a unique and suitable system for investigating certain complexities of the human AS phenotype, particularly social communication and affect (Brudzynski, 2013; Wöhr and Schwarting, 2013; Burke et al., 2017; Homberg et al., 2017; Braun et al., 2018; Fernández et al., 2018; Burgdorf et al., 2020; Netser et al., 2020). Our discovery of excessive laughter-like 50 kHz USV is the first report of this affective outcome measure in a model of AS, mirroring the affected population. Moreover, reduced social play, atypical gait, impaired cognition, and anatomic and cellular physiology anomalies were easily detected in this model.

We leveraged our model species to discover that *Ube3a*^mat–/pat+^ rats produced an overabundance of 50 kHz vocalizations, which reflect a positive affective state and have been referred to as rat laughter (Panksepp and Burgdorf, 2000; Panksepp, 2005; Rygula et al., 2012), as well as a trend of elevated laughter-like 50 kHz USV without provocation. Excessive 50 kHz USV, suggestive of enhanced “wanting” and “liking” the interaction (Berridge, 2009; Berridge and Aldridge, 2009; Berridge et al., 2009; Okabe et al., 2021), closely aligns with the AS profile of a happy disposition and easily provoked laughter. To our knowledge, this is the first report of this method being used in a genetic rat model of a neurological disorder.

Exaggerated 50 kHz calling could suggest enhanced effort to elicit social interaction or may be unrelated to the social component of heterospecific play, potentially a neurobiological consequence of a disinhibited vocal production pathway. AS is typified by laughter that is easily provoked regardless of stimuli valence. The phenotype may also reflect enhanced sensitivity to tactile stimulation. Deriving greater reward from physical interactions could explain typical levels of social investigation in the reciprocal interaction test but reduced social approach in the previously reported three-chambered and USV playback assays, as well as the disinhibition of social interactions in the clinical population. One limitation of our USV analysis was the lack of acoustic feature quantification for the calls evoked by heterospecific play. We did, however, subsequently perform this analysis for all other USV assays and found no genotype effect on call features.

Juvenile social play is a critical way that rats develop social competence and learn how to appropriately engage and communicate with others, analogous to play in young children (Hofer and Shair, 1978; Panksepp and Beatty, 1980; Panksepp, 1981; Brudzynski, 2009, 2013; Argue and McCarthy, 2015). *Ube3a*^mat–/pat+^ rats were interested in a novel partner but did not engage in rough-and-tumble play behaviors characteristic of the species, albeit specific to sex and strain. Our finding of no sex difference in rough-and-tumble play aligns with previous reports, which also used pretest social isolation to motivate the subjects to play (Veenema et al., 2013; Bredewold et al., 2014, 2015; Reppucci et al., 2018; Kisko et al., 2020). In contrast with studies on mouse models of AS using the three-chambered social approach task (Jamal et al., 2017; Kumar et al., 2019; Dutta and Crawley, 2020; Perrino et al., 2021), which have reported contradictory social deficits and “hypersociability,” the rat model displayed a typical level of social investigation.

Movement disorders (Wheeler et al., 2017) are a hallmark feature of AS, with gait ataxia being one of the most common issues. While the deficits of *Ube3a*^mat–/pat+^ rats were not obvious to the eye, subtle aberrations in stance and paw placement, paired with abnormal braking and propelling, reflect impaired motor coordination. All of this evidence suggests that altered postures affect motor dynamics, which results in the gait patterns exhibited by AS individuals and *Ube3a*^mat–/pat+^ rats. The limb weakness indicated by our gait analysis aligns with the reduced rearing previously observed (Berg et al., 2020c).

We discovered and report for the first time, to our knowledge, LTP deficits in this rat model (Jiang et al., 1998; Weeber et al., 2003; van Woerden et al., 2007; Filonova et al., 2014; Ciarlone et al., 2016), which provides a putative cellular signaling mechanism underlying the learning and memory impairments reported herein and previously (Berg et al., 2020c; Dodge et al., 2020). Juvenile *Ube3a*^mat–/pat+^ rats exhibited deficits in cued fear memory 48 h after training, which extends the previous finding by Dodge et al. (2020) of deficient contextual and cued fear conditioning in adults 72 h after training. We ruled out impaired sensorimotor abilities as a confounding variable since the acoustic startle response was unaffected.

Pronounced deficits in adulthood are supported by neuroimaging. Previously, we discovered a variety of trending volumetric abnormalities at PND 21 (Berg et al., 2020c); however, these new data show more substantial reductions in adults throughout the brain, highlighting a more severe neuroanatomical phenotype with age. Reduced total brain volume may indicate a loss of cellular volume or dendritic complexity over time, and the drastic volume loss in fiber tracts could indicate a loss in axonal numbers, axonal volume, or myelination. In a mouse model of AS, white matter loss was found to play a large role in the overall microcephaly observed (Judson et al., 2017), with an 11% loss in the corpus callosum making it the most affected white matter structure. *Ube3a*^mat–/pat+^ rats showed a trend toward reduced corpus callosum volume (−6.7%), but the largest white matter deficits were cerebellar. In alignment with Judson et al.'s (2017) study in mice, the reduced fiber tract volume was also disproportionate to the overall brain volume loss, confirming that white matter development plays the major role in the impaired brain growth in AS. Additionally, the 9% decrease in cerebellum size was consistent with cortical loss (−9%), but there was a disproportionate reduction in arbor vitae and deep cerebellar nuclei volume, indicating that the outputs of the cerebellum are impaired.

GABAergic neuron loss (Judson et al., 2016) and decreased tonic inhibition in cerebellar granule cells (Egawa et al., 2012) underlie the theory of brain dysfunction in AS, and hypotheses addressing the theory of reduced inhibitory tone are being pursued for small-molecule development (Ciarlone et al., 2017). The present results are congruent with loss of inhibitory tone as the overarching mechanism theory of AS. Emission of 50 kHz USVs induced by heterospecific play is associated with dopamine release in the nucleus accumbens/ventral striatum (Hori et al., 2013), as is reception of 50 kHz USV (Willuhn et al., 2014). These calls have been considered as signals of “joy,” “euphoria,” and “laughter,” supported by behavioral pharmacology showing increased 50 kHz USVs resulting from amphetamine administration (Brudzynski, 2013, 2015; Wöhr, 2021) and electrical stimulation of reward-associated areas (Burgdorf et al., 2007). Both vocalizations and gait require fine motor control; thus, striatal and motivational components support aberrant frontal-striatal circuitry. Interestingly, the ventral striatum and “reward” associated substrates of the basal ganglia have inhibitory projections, in line with overall theories of AS regarding inhibitory loss.

While gross and fine movement are complicated multisystem physiological processes, AS individuals show ataxic movements in both upper and lower limbs and aberrant gait, suggesting particular involvement of the cerebellum. This was corroborated here and in earlier work by the large reductions in cerebellar nuclei size, which is consistent with our overarching mechanistic hypothesis since Purkinje cell neurons projecting to the deep cerebellar nuclei modulate excitation via inhibition and Egawa et al. (2012) highlighted decreased cerebellar granule cells in AS. Using conditional *Ube3a* mouse models to identify the neural substrates of circuit hyperexcitability in AS, Judson et al. (2016) provided compelling evidence that GABAergic, but not glutamatergic, *Ube3a* loss is responsible for mediating the EEG abnormalities and seizures of AS. Previously, we reproduced and extended the Judson et al. (2016) data (Copping and Silverman, 2021), and our hypothesis is that this mechanism extends to social communication, cognitive phenotypes, and impaired gait outcomes, as each shares components of learning and motivation. The loss of brain volume in regions dense with inhibitory neurons seen herein provides further corroborative evidence that GABAergic tone underlies functional outcomes. Independent corroboration comes from ErbB inhibitors, which have been reported to reverse LTP deficits in AS model mice (Kaphzan et al., 2012). While glutamate receptor expression and function were unaltered in *Ube3a* mice (Kaphzan et al., 2012; Judson et al., 2016), ErbB signaling was shown to rescue LTP impairments in *Ube3a* mice via an increase in inhibitory synaptic transmission, corroborating our core overarching mechanism of reduced inhibitory tone.

In conclusion, we discovered that *Ube3a*^mat–/pat+^ rats exhibited interest in a social partner but expressed an atypically high level of laughter-like vocalizations. Deficits in other AS-relevant domains were also discovered, including gait and cognition, and reduced hippocampal LTP. Future lines of investigation will assess the circuitry and mechanisms underlying the excessive laughter-like USV and social-cognitive anomalies in USV reception, in addition to pursuing other neurobiological endpoints. Overall, our results indicate that the deletion of maternal *Ube3a* in the rat creates a sophisticated rodent model with high face validity to the human AS phenotype. In the pursuit of effective therapeutics, it is essential to be equipped with a diverse set of behavioral outcome measures and neurologic biomarkers by which to assess efficacy. Together, we demonstrate that the *Ube3a*^mat–/pat+^ rat offers numerous potential outcome measures that are detectable throughout the lifespan.
